# Co-Circulation of *Leishmania* Parasites and Phleboviruses in a Population of Sand Flies Collected in the South of Portugal

**DOI:** 10.3390/tropicalmed9010003

**Published:** 2023-12-20

**Authors:** Fátima Amaro, Anabela Vilares, Susana Martins, Tânia Reis, Hugo Costa Osório, Maria João Alves, Maria João Gargaté

**Affiliations:** 1 Centre for Vectors and Infectious Diseases Research, National Institute of Health Doutor Ricardo Jorge, No. 5, 2965-575 Águas de Moura, Portugal; hugo.osorio@insa.min-saude.pt (H.C.O.); m.joao.alves@insa.min-saude.pt (M.J.A.); 2 Environment and Infectious Diseases Research Group, Environmental Health Institute, 1649-028 Lisboa, Portugal; 3 Centre for Animal Science Studies (CECA), University of Porto, 4050-453 Porto, Portugal; 4 National Reference Laboratory of Parasitic and Fungal Infections, National Institute of Health Doutor Ricardo Jorge, 1649-016 Lisboa, Portugal; anabela.vilares@insa.min-saude.pt (A.V.); susana.martins@insa.min-saude.pt (S.M.); tania.reis@insa.min-saude.pt (T.R.); m.joao.gargate@insa.min-saude.pt (M.J.G.)

**Keywords:** Phleboviruses, Phlebotomine sand flies, *Leishmania* spp., Portugal

## Abstract

In the Old World, phlebotomine sand flies from the genus *Phlebotomus* are implicated in the transmission of *Leishmania* spp. parasites (Kinetoplastida: Trypanosomatidae) and viruses belonging to the genus *Phlebovirus* (Bunyavirales: Phenuiviridae). Two of the five sand fly species known to occur in Portugal, *Phlebotomus perniciosus* and *Ph. ariasi*, the former being the most ubiquitous, are recognized vectors of *Leishmania infantum*, which causes visceral leishmaniasis, the most prevalent form of leishmaniasis in the country. *Phlebotomus perniciosus* is also the vector of the neurotropic Toscana virus, which can cause aseptic meningitis. Entomological surveillance is essential to provide fundamental data about the presence of vectors and the pathogens they can carry. As such, and given the lack of data in Portugal, an entomological survey took place in the Algarve, the southernmost region of the country, from May to October 2018. Polymerase chain reaction assays were performed in order to detect the presence of the above-mentioned pathogens in sand fly pools. Not only were both *Leishmania* parasites and phleboviruses detected during this study, but more importantly, it was the first time their co-circulation was verified in the same sand fly population collected in Portugal.

## 1. Introduction

Phlebotomine sand flies (Diptera, Psychodidae) are insects with worldwide distribution. Despite their small size, they are very important in terms of spreading infectious diseases. With few exceptions, the females require a blood meal to produce eggs. They can acquire pathogens while feeding on infected hosts and transmit them to other hosts during the next blood meal. Sand flies of the genus *Phlebotomus* are vectors of flagellated obligate intracellular protozoans of the genus *Leishmania* (Kinetoplastida: Trypanosomatidae) and of viruses belonging to the genus *Phlebovirus* (Bunyavirales: Phenuiviridae) in several countries of the Old World [1].

The taxonomy of the genus *Leishmania* has been the subject of much debate and is a controversial topic, making it difficult to differentiate between species [2]. Vickerman (1976) proposed the recognition of four species complexes within the genus: the *donovani* complex, the *tropica* complex, the *mexicana* complex, and the *braziliensis* complex (adapted later, partially by Lainson and Shaw) [3,4]. The clustering of *Leishmania* at the subgeneric level and the definition of “complexes” in the *Leishmania* classification have gained rather wide acceptance, but there are still serious challenges in terms of the genus composition. In this way, defining a *Leishmania* species or accepting all the described species is still not straightforward [5]. Notwithstanding, four species of *Leishmania* are recognized in Europe and surrounding countries: *L. donovani* complex species, which includes both *L. infantum* and *L. donovani* s.s.; *L. tropica* and *L. major* [6]. *Leishmania infantum* is distributed throughout southern European countries. To date, *L. donovani* s.s. has only been described in Turkey and Cyprus, and *L. major* and *L. tropica* are limited to northern Africa and in some parts of the Caucasus [6,7,8]. In addition, there is evidence of the presence of *L. tropica* in Crete during the first decade of the twenty-first century, implying the disease had re-emerged on that island [6,9].

Leishmaniasis is the designation for the disease caused by *Leishmania* parasites. It can manifest in three main forms: visceral (VL), often known as kala-azar and although less incident, the most severe form; cutaneous (CL), the most common; and mucocutaneous (MCL), the most disabling form of the disease. Visceral leishmaniasis, caused by *L*. *infantum,* is mainly transmitted by *Ph. perniciosus* and *Ph. ariasi* sand flies and is endemic in Europe and the Mediterranean basin [10,11,12,13]. The VL was mostly known as a pediatric disease, although in the last few years, a decrease in cases has been observed, while an increase in infection in adults has been noted, usually associated with HIV/AIDS. This seems to be a common trend in countries in the south of Europe, including Portugal [10,11]. Based on the World Health Organization’s (WHO) Global Health Observatory Data Repository (GHDR), after reaching a value of 0.4 from 2013 to 2016 in Portugal, the median annual incidence of VL per 100,000 inhabitants increased to 0.06 in the period 2017−2020 [6]. There are, however, discrepancies in the reported incidence between WHO sources and hospital discharge records available in some countries. In this way, and due to the underreporting of leishmaniasis cases, there is an urgent need to improve surveillance and notification systems [6].

In Portugal, there are three recognized leishmaniasis foci: the Alto Douro region in the north, the Algarve in the south, and suburban areas of Lisbon [10]. Besides being a human threat, *L. infantum* also poses a serious veterinary problem since canine leishmaniasis (CanL) commonly presents a fatal outcome. The CanL is increasing in Portugal and is recorded with a prevalence of 20% in endemic foci [10]. Due to the mode of infection (through sand fly bites), it is easily transmitted to other dogs or humans. Dogs are considered the main reservoirs for human VL, and, for that reason, in order to prevent the spread of human transmission, infections in these domestic animals should be contained [14,15].

Phleboviruses are single-stranded RNA viruses with a tri-segmented genome composed of small (S), medium (M), and large (L) segments [16]. The taxonomy of phleboviruses is constantly changing, and currently, the International Committee on Taxonomy of Viruses (ICTV) recognizes 67 phleboviruses species [17]. In Europe and in the Mediterranean region, the most medically important phlebovirus transmitted by sand flies is Toscana virus (TOSV) (recently renamed as *Phlebovirus toscanaense*), which can cause not only asymptomatic or mild flu-like syndromes but also self-limiting neuro-invasive diseases such as meningitis or meningoencephalitis. This virus has been considered the third main cause of aseptic meningitis in the Mediterranean region during the summer months and, as such, a major public health concern [18]. Furthermore, TOSV has been associated with unusual clinical manifestations, with or without permanent sequelae, and even with some cases of fatal outcomes [19,20,21]. Although human seroprevalence studies for TOSV show numbers around 10–24%, in endemic regions such as Tuscany, this value can go up to 77% [19,22].

The proven vectors of TOSV are *Ph. perniciosus* and *Ph. perfiliewi* [23]. Other circulating phleboviruses in the Mediterranean region associated with human disease, namely some members of the sand fly fever Sicilian virus (*Phlebovirus siciliaense,* SFSV)*,* including Sicilian, Turkish, and Cypriot variants and the sand fly fever Naples virus species (*Phlebovirus napoliense*) are also transmitted by sand flies of the *Larroussius* group (e.g., *Ph. ariasi*, *Ph. papatasi*). These phleboviruses may cause febrile syndromes accompanied by headaches, malaise, photophobia, myalgia, and retro-orbital pain, usually known as sand fly fever, three-day fever, or pappataci fever [24]. Nowadays, it is believed that phlebovirus infections are largely underreported, and this may happen due to the number of asymptomatic or mild disease cases.

To date, at least four phleboviruses are known to circulate in Portugal: TOSV with reported isolation and serological evidence in humans and serological evidence in cats and dogs; Massilia and Alcube viruses (*Phlebovirus massiliaense* and *Phlebovirus alcubeense*, respectively), isolated and detected, up to date, only in sand flies; and the SFSV recently associated with human disease in Portugal and detected in sera samples of humans, cats, and dogs [25,26,27,28,29,30,31,32,33,34].

In the most recent study conducted to evaluate the presence of antibodies against TOSV and SFSV in a human population residing in the southwest of Portugal, seroprevalences of 5.3% and 4.3% were detected, respectively [32]. Nonetheless, phlebovirus infections are still neglected in the country.

There are five species of sand flies currently known to circulate in Portugal: *Ph. ariasi*, *Ph. papatasi*, *Ph. perniciosus*, *Ph. sergenti*, and *Sergentomyia minuta.* Interestingly, in this country, although *Leishmania* parasites have been detected several times in their natural vectors, to date, the only phleboviruses locally recognized as causes of disease (TOSV and SFSV) have never been isolated in sand flies. Moreover, data regarding this group of insects are largely limited in Portugal. If we want to be prepared for outbreaks and promptly engage in public health interventions, information about the circulating vectors and the pathogens they can carry and transmit is essential. In this way, entomological surveillance is of utmost importance and should be consistently implemented.

The aim of this work is to report the findings of an entomological survey performed in the Algarve region, Portugal, during the summer of 2018, where we found, for the first time in our country, the co-circulation of *Leishmania* parasites and phleboviruses in wild-caught sand flies.

## 2. Materials and Methods

### 2.1. Field Survey

The study was conducted in Algarve, south of Portugal, from May to October 2018. The area covered by the survey is characterized mainly by a temperate climate with a rainy winter (precipitation concentrated between October and April) and a hot, dry summer [35].

Sand flies were trapped with modified (ultra-fine mesh) CDC light traps (John W. Hock Company, Gainesville, FL, USA) baited with dry ice, placed at sunset, and collected after sunrise, during four consecutive nights. The traps were positioned in selected locations of known ecological preferences of the sand flies (e.g., animal facilities with organic matter and high humidity, such as kennels, rabbit, and chicken pens). After collection, the sand flies were stored at −80° until further processing in the laboratory.

### 2.2. Identification of Sand flies

Once in the laboratory, around 10% of the male sand flies were mounted on spot slides for morphological identification to species level in a stereomicroscope according to existing taxonomic keys [36,37]. Regarding female sand flies, when there was only one specimen per pool for the pathogen screening, they were identified by molecular methods using the cytochrome c oxidase subunit I gene of mitochondrial DNA, as previously described [38].

### 2.3. Phleboviruses and Leishmania spp. Detection and Sequencing

All female sand flies were processed in pools for detection of phleboviruses and *Leishmania* parasites. Male sand flies not used for morphological identification were also processed in pools for detection of phleboviruses.

Pools of one to 35 sand flies were organized by sex, date, and place of collection and were grounded and suspended in Hank’s solution containing 7.5% bovine albumin and antibiotics, as previously described [28]. After centrifugation of the mixture, a 400 μL aliquot of the supernatant fluid was used for pathogen detection, and the remainder was stored at −80° for further viral isolation attempts and other studies.

For detection of phleboviruses, after nucleic acid extraction (NUCLISENS^®^ easyMAG^®^, bioMérieux, Marcy-l’Étoile, France), a pan-phlebo rt-PCR targeting a 370-nucleotide region of the S segment of phleboviruses was performed [39]. In the case of positive samples, pools were used to infect VERO E6 cells as previously described [39]. For the detection of *Leishmania*, and after nucleic acid extraction as mentioned above, pools of female sand flies were screened by nested PCR amplification of the 18S rRNA gene [13,40]. Despite whole-genome sequencing and multilocus sequence typing (MLST) being the traditional methods used to differentiate between the species of the *L. donovani* complex, the 18S PCR is the most sensitive for identifying *Leishmania* spp. The choice of this PCR marker is consistent with a study by León et al. in which the authors reported that the 18S rRNA marker exhibited the best performance in terms of analytical sensitivity and specificity for the detection of *Leishmania* spp. [41]. Also, it is widely acknowledged that many regions of the 18S rRNA gene are either completely conserved or partially conserved [42].

All PCR products (from the sand fly species identification and both pathogen reactions) were visualized in GelRED (Biotarget, Lisbon, Portugal)-stained 1.5% agarose gel electrophoresis, purified, and sequenced in an ABI 3130xl Genetic Analyzer (Applied Biosystems, Foster City, CA, USA).

Homology searches were performed using the BLAST algorithm [43], and partial sequences were aligned with control sequences retrieved from GenBank via Clustal W in Bioedit [44].

## 3. Results

### 3.1. Collected Sand Flies

**Figure 1:**
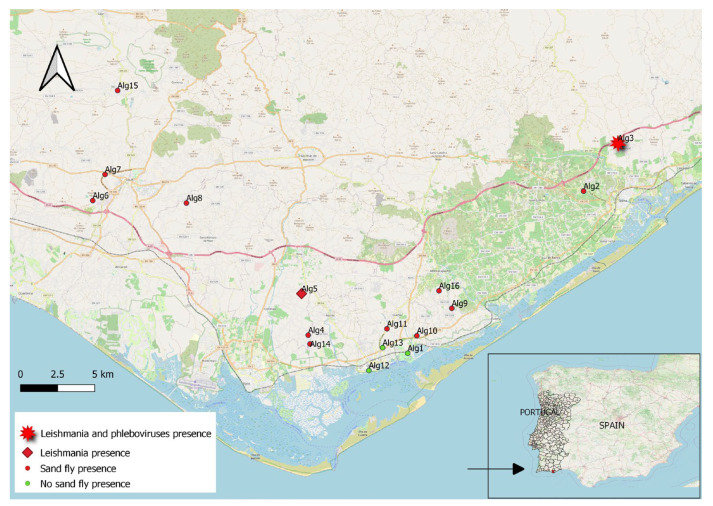
Surveyed sites and collection of sand flies; the location of the co-circulation sites (*Leishmania* and phleboviruses) are also shown.

Sixteen field stations distributed in nine parishes belonging to the counties of Loulé, Olhão, and Tavira were surveyed. Sand flies were found in 13 of 16 field stations (Figure 1).

A total of 1161 specimens were collected, comprising of 736 females and 425 males. One hundred and eighteen specimens were identified to species level, and four species were found: *Ph. ariasi*, *Ph. perniciosus*, *Ph. sergenti*, and *Se. minuta* (Table 1).

### 3.2. Detection of Phleboviruses and Leishmania *spp.*

We tested 736 females organized in 140 pools and 394 males distributed in 38 pools (Table 1).

The RNA of two phleboviruses – PoSFPhlebV/11/2018 and PoSFPhlebV/38/2018 – was detected in pools 11 and 38 (each one composed of 20 female sand flies), respectively, in a rural setting in Tavira, Santa Maria parish (Alg3), in a hennery adjacent to a kennel in May and June 2018. Isolation attempts were not successful, but partial genome sequences of the nucleoprotein gene (segment S) were obtained, and these were deposited in GenBank. For PoSFPhlebV/11/2018, a sequence of 381 nucleotides was retrieved (ON807199), phylogenetically distinct from other known phleboviruses. The other obtained sequence (530 nucleotides, ON807200) seems to belong to a Massilia virus strain. These findings were all previously published, including the sequence files and the respective phylogenetic trees, and can be found in Amaro et al. [19].

Regarding *Leishmania* parasites, DNA was detected in six pools: four in Tavira, Santa Maria parish (Alg3), in May and June (pools 19, 45, 57, and 61), at the exact location as above mentioned, and two in Conceição, Olhão (Alg5) in June (pools 32 and 86), near a rabbit hutch, in a pedagogical farm (Table 2). Three sequences were deposited in GenBank (accession numbers: OR783264-66). After alignment analysis, the sequences were attributed to the *L. donovani* complex since the sequencing of these PCR products did not allow for species discrimination. Sequencing of pools 57, 61, and 86 was not possible due to low DNA concentration, but we assumed they belonged to the same complex found in the other pools.

The co-circulation of phleboviruses and species from the *L. donovani* complex in the same sand fly population was detected on 16 May and 5 June, 2018 in Tavira (Alg3). The location of the surveyed sites and detected pathogens is depicted in Figure 1 (geographical coordinates are available in Supplementary Table S1). Data regarding the detections are presented in Table 2.

## 4. Discussion

Four of the five species of sand flies known to occur in Portugal were collected during this survey. This is in accordance with previous studies [45,46]. *Phebotomus perniciosus* was the most identified species in our samples and was present in all the sites where sand flies were found. This conforms with other surveys where it is referred to as the most ubiquitous species in Portugal, present from north to south [47]. *Phlebotomus ariasi,* another vector of *L. infantum* responsible for zoonotic visceral leishmaniasis in western Mediterranean countries, was collected in two localities (Santa Maria and São Sebastião). This species has been shown to be dominant in some regions of Portugal, namely in Douro [48]; however, that was not the case in our study. *Phlebotomus papatasi*, the only missing species in our survey (from the five known to exist in the country), is considered uncommon in Portugal [37].

Two phleboviruses were detected in our study. One of them may be an unknown phlebovirus, at least in Portugal, but it was not possible to confirm it further with genomic analysis. Nevertheless, the sequence obtained from the standard nucleotide BLAST (blastn) [43] presented similarities with *Phlebovirus hediense* [49], which is a largely unknown phlebovirus, previously isolated from sand flies in China, and with phleboviruses collected in Italy, with only partial sequences available in GenBank. The other detected phlebovirus seems to be a Massilia virus strain since it clustered together with other strains isolated or detected previously in the south of Portugal [19]. However, the partial phleboviruses sequences obtained in the Algarve in 2018 are too small to make a valid assumption. We would need to perform whole genome sequencing to be certain of the identity of those phleboviruses, specifically with the complete sequences of the RdRp gene (Large segment). Currently, the ICTV uses criteria to differentiate phleboviruses’ species [17]. These findings were already discussed in a previous publication [19]. Still, we can hypothesize that at least two phlebovirus species were in circulation in the same location at that time. The co-circulation of different phleboviruses is not uncommon. It was reported previously in Portugal and should not go unnoticed as recombination events may occur, given the genetic characteristics of these viruses, which could result in new strains or species with unknown pathogenic potential [29].

The sand fly pools where both phleboviruses were detected were composed of 20 female specimens each. Moreover, at that collection site (Alg3), three sand fly species were identified: *Ph. ariasi*, *Ph. perniciosus*, and *Se. minuta*. Although it seems that *Ph. perniciosus* is the most abundant species at that location, we cannot make any conjectures about the sand fly species with which the referred phleboviruses might be associated. However, the first reported isolation of the Massilia virus was achieved in pools of *Ph. perniciosus* sand flies collected in 2005 in Marseille, France, and this may indicate the species as the probable vector for the referred virus [50].

Concerning *Leishmania* detections, species of the *L. donovani* complex were identified. We can assume that all the isolates belong to the same complex and further hypothesize that we are in the presence of *L. infantum*, a fact supported by previous studies that recognized this species as the only one circulating in Portugal and the most prevalent in southern Europe [6,12,51,52,53].

The pools found positive for *Leishmania* parasites were all composed of a unique sand fly specimen. Although *Ph. ariasi* is also one of the vectors of the *L. donovani* complex reported as naturally infected with *Leishmania* in Portugal [51], and specimens of this species were identified during this study, all the sand flies found positive for this pathogen were identified as *Ph. perniciosus*. Given that *L. infantum* has been mostly detected in the latter species of sand fly in our country, this result is not surprising [46,51]. In fact, *Ph. perniciosus* was considered the primary vector of *Leishmania* parasites in the region in a former study because, similarly to the present work, it was not only the one species found infected with *L. infantum* but also the one that presented the highest abundance and distribution [52].

The co-circulation of *L. infantum* and phleboviruses in sand fly populations in the Mediterranean region has been reported in preceding studies. Fares et al. documented the endemic co-circulation of TOSV and that parasite in a zoonotic VL focus in Central Tunisia [54]. Moreover, Ergunay and colleagues, in research conducted in the eastern Thrace region of Turkey and Northern Cyprus, found a pool of *Ph. tobbi* co-infected with TOSV and *L. infantum* [55]. Different sand fly pools were found infected with both the Massilia virus and *L. infantum* at the same trapping site in an urban area in Marseille [56]. Furthermore, Calzolari and colleagues reported the detection of *Leishmania* and phleboviruses in the same pools, namely, Fermo virus (14 pools), Ponticelli virus (1 pool), and Corfou virus (1 pool) [57]. All these findings lead us to hypothesize that contemporaneous infections in local sand fly populations may be translated into the transmission of both pathogens.

In this study, we report, for the first time in Portugal, the circulation of *Leishmania* and phleboviruses in wild-caught sand fly populations. However, we did not find both pathogens in the same insect pool. Even though Dincer et al. assume that there is a low probability of double infections in sand flies [58], we believe that, to date, there are not enough data to exclude the possibility of co-infected sand flies transmitting both agents. As such, further investigation is required, and experimental co-infections of vector sand flies reared in laboratory colonies may further help to unravel some of these questions.

The co-infection of hosts with phleboviruses and *Leishmania*, on the other hand, has been the subject of several laboratory studies. Rossi and colleagues reported the findings of an exacerbated murine leishmaniasis disease when laboratory mice were co-infected with *L. guyanensis* and TOSV [59]. The latter virus favored the parasite’s persistence and spreading. In 2019, Rath et al. reported the use of *Phlebovirus icoaraciense*, isolated from the rodent *Nectomys sp*., which is also a sylvatic reservoir of *L. amazonensis*, and demonstrated that the co-infection of both pathogens boosted the parasite load, both in vivo and in vitro [60]. Likewise, Heirwegh and colleagues (2021), using wild-type and knock-out mouse models, determined that co-infection with *L. major* and SFSV influenced disease severity, inducing aggravated skin lesions and higher parasite numbers [61].

Regarding natural co-infections of vertebrate hosts, Dincer et al. documented for the first time the co-infection of TOSV and *L. infantum* in two dogs in Adana province, Turkey [58]. These dogs presented clinical symptoms compatible with canine leishmaniasis, namely anorexia, wasting, muscle atrophy, epistaxis, and mucosal bleeding. Furthermore, several serological studies demonstrated the presence of antibodies against *Leishmania* and phleboviruses, and it has been suggested that dogs infected with *L. infantum* are extremely appealing to *Ph. perniciosus* when compared to healthy ones [62]. For that reason, they may serve as sand fly magnets and promote a substantial increment in the contact between the vector and infected dogs. Moreover, in a study aimed at investigating TOSV infections in healthy and infected dogs with *Leishmania* following natural exposition to sand fly bites in Northern Tunisia, the authors demonstrated for the first time that, in addition to their role as the main reservoir hosts of *L. infantum*, dogs are competent reservoirs for the transmission of TOSV to sand fly vectors in natural settings [63]. Likewise, in Portugal, in a seroprevalence study in two different districts in the south, a significant association was observed between the presence of antibodies to *L. infantum* and SFSV in sheltered dogs [64].

Concerning human co-infections, Bichaud and colleagues were able to find a significant correlation between the seropositivity of *L. infantum* and TOSV in a retrospective serological screening in southern France and delivered the first strong indication for the existence of an epidemiological relationship between both infections [65]. Following this line of thought, Heirwegh et al., while studying severe cases of CL, pointed out that to improve patient outcomes and the time to disease clearance, the possibility of phleboviral co-infections should be considered and the disease treated accordingly [61]. Even if there is presently no evidence of co-infection in the vectors, the proven co-circulation of the pathogens in the same population of sand flies is enough to raise our attention and supports the idea that co-infections should be considered in vertebrate hosts. Regarding the Mediterranean region in particular, further research is essential in order to understand the implications of *L*. *infantum* and phleboviruses’ co-infections.

## 5. Conclusions

The co-circulation of *Leishmania* parasites and phleboviruses in the same population of sand flies is documented here for the first time in Portugal. More research is needed to better understand the impact of these co-infections, not only in the vector populations but also in the susceptible hosts who may develop the disease.

## Figures and Tables

**Table 1:** Sand flies collected and identified by sampled site and month of collection.

Collection Site	Parish, Municipality/Month	Number of Collected Sand Flies	Total Number of Studied Pools (f/m)	Identified Species
Alg1	Quelfes, Olhão/May	0	-	-
Alg2	Santiago, Tavira/May	13	7 (6/1)	*Ph. perniciosus* (3f; 1m)
Alg3	Santa Maria, Tavira/May, June	438	57 (48/9)	*Ph. ariasi* (2f; 2m), *Ph. perniciosus* (25f; 7m) *Se. minuta* (1f)
Alg4	Conceição, Olhão/June	50	12 (1/11)	*Ph. perniciosus* (7f)
Alg5	Conceição, Olhão/June	57	12 (9/3)	*Ph. perniciosus* (5f; 1m)
Alg6	Loulé, Loulé/July	3	2 (0/2)	*Ph. perniciosus* (1f)
Alg7	São Sebastião, Loulé/July	78	16 (13/3)	*Ph. ariasi* (1f), *Ph. perniciosus* (7f; 2m)
Alg8	Santa Bárbara de Nexe, Loulé/July	3	18 (12/6)	*Ph. perniciosus* (1f)
Alg9	Moncarapacho, Olhão/August	85	16 (10/6)	*Ph. perniciosus* (3f; 3m)*, Ph. sergenti* (1f)
Alg10	Quelfes, Olhão/August, October	229	25 (17/8)	*Ph. perniciosus* (10f; 8m)
Alg11	Quelfes, Olhão/August	15	3 (2/1)	*Ph. perniciosus* (1m)
Alg12	Olhão, Olhão/September	0	-	-
Alg13	Quelfes, Olhão/September	0	-	-
Alg14	Conceição, Olhão/September	98	8 (6/2)	*Ph. perniciosus* (2f; 4m)
Alg15	Tôr, Loulé/October	24	8 (6/2)	*Ph. perniciosus* (3f; 2m)*, Ph. sergenti* (1f)
Alg16	Moncarapacho, Olhão/September, October	68	10 (8/2)	*Ph. perniciosus* (5f; 1m)

f—female; m—male.

**Table 2:** Collection stations, sampling dates, and pathogen identification in the sand fly pools.

Collection Station	Sampling Date in 2018	Pool Size (f)/#Pool Number	DNA Barcoding	Identified Pathogen/GenBank Accession Number	Reference
Alg3	16 May	20/#11	-	New phlebovirus?/ON807199	[19]
Alg3	16 May	1/#19	*Ph. perniciosus*	*L. donovani* complex OR783264	This work
Alg5	5 June	1/#32	*Ph. perniciosus*	*L. donovani* complex *OR783265*	This work
Alg3	5 June	20/#38	*-*	Massilia phlebovirus/ON807200	[19]
Alg3	5 June	1/#45	*Ph. perniciosus*	*L. donovani* complex * *OR783266*	This work
Alg3	7 June	13/#57	*-*	*L. donovani* complex *	This work
Alg3	7 June	1/#61	*Ph. perniciosus*	*L. donovani* complex *	This work
Alg5	7 June	1/#86	*Ph. perniciosus*	*L. donovani* complex *	This work

f—female; *—Sequencing was not possible due to low DNA concentration.

## Data Availability

Data are contained within the article and Supplementary Materials.

## Supplementary Materials

The following supporting information can be downloaded at: https://www.mdpi.com/article/10.3390/tropicalmed9010003/s1, Table S1, Geographical coordinates of the surveyed sites.
