# Supplementary material for: Co-Circulation of Leishmania Parasites and Phleboviruses in a Population of Sand Flies Collected in the South of Portugal
Source: Trop Med Infect Dis. 2023 Dec 20;9(1):3. doi: 10.3390/tropicalmed9010003 (PMC10821132; doi:10.3390/tropicalmed9010003)
Supplement: Supplementary file 1 [file tropicalmed-09-00003-s001.zip › tropicalmed-2757475-supplementary.pdf]

Supplementary Table S1: Geographical coordinates of the surveyed sites

Colllection site	Latitude	Longitude	Municipality	Parish	Tipe of animaL facility	Sand fly presence	Detected pathogens
Alg1	37.034308	-7.813126	Olhão	Quelfes	Wildlife recovery centre	No	
Alg2	37.132019	-7.679487	Tavira	Santiago	Chicken hen	Yes	
Alg3	37.161070	-7.653302	Tavira	Santa Maria	Chicken hen	Yes	*Leishmania* and phleboviruses
Alg4	37.045441	-7.888368	Olhão	Conceição	Chicken hen	Yes	
Alg5	37.070583	-7.893326	Olhão	Conceição	Rabbit hut in a pedagocical farm	Yes	*Leishmania*
Alg6	37.127178	-8.051329	Loulé	Loulé	Animal shelter (dogs, cats, donkeys)	Yes	
Alg7	37.142954	-8.042016	Loulé	S.Sebastião	Chicken hen	Yes	
Alg8	37.125607	-7.980422	Loulé	S. Bárbara Nexe	Horse enclosure	Yes	
Alg9	37.061411	-7.779654	Olhão	Moncarapacho	Kennel	Yes	
Alg10	37.044877	-7.806156	Olhão	Quelfes	Chicken hen	Yes	
Alg11	37.049138	-7.828777	Olhão	Quelfes	Kennel	Yes	
Alg12	37.023950	-7.842566	Olhão	Olhão	Pigeonry	No	
Alg13	37.037715	-7.832078	Olhão	Olhão	Chicken hen	No	
Alg14	37.040103	-7.887072	Olhão	Conceição	Kennel	Yes	
Alg15	37.193799	-8.032382	Loulé	Tôr	Chicken hen	Yes	
Alg16	37.072063	-7.789237	Olhão	Moncarapacho	Kennel	Yes	
